# Egg intake and bladder cancer risk: A meta-analysis

**DOI:** 10.3892/etm.2012.671

**Published:** 2012-08-16

**Authors:** DANBO FANG, FUQING TAN, CHAOJUN WANG, XUANWEN ZHU, LIPING XIE

**Affiliations:** Department of Urology, The First Affiliated Hospital, School of Medicine, Zhejiang University, Hangzhou, Zhejiang 310003, P.R. China

**Keywords:** eggs, diet, bladder cancer, meta-analysis

## Abstract

Egg intake has been hypothesized to promote carcinogenesis due to its potential to increase circulating levels of cholesterol. Epidemiological findings regarding the association between egg consumption and risk of bladder cancer have been inconsistent. We performed a meta-analysis of the available data. Relevant studies were identified by a PubMed database search of articles dating from between January 1980 and December 2011. We identified 4 cohort and 9 case-control studies of egg intake and risk of bladder cancer. Both fixed- and random-effects models were used to calculate the summary risk estimates (REs). The combined RE of bladder cancer for the highest compared with the lowest egg intake was 0.94 (95% CI, 0.69–1.18) and weak evidence of heterogeneity was observed. The association between egg intake and risk of bladder cancer differed significantly by geographic region, with a 28% reduced risk in Japanese. Our results provided no strong evidence of a significant association of egg consumption with bladder cancer incidence but showed a protective effect in Japanese.

## Introduction

Bladder cancer is the fourth most common cancer among men in the United States. In 2010, it was estimated that 69,250 new cases of bladder cancer would be diagnosed and 14,990 deaths of this cancer would occur among men in the country (1). To date, the only firmly established risk factors for this disease appear to be age, gender, smoking history and occupational exposure to certain chemicals (2). However, these well-defined factors cannot fully explain the observed differences in incidence and mortality from bladder cancer amongst countries. Therefore, other potential risk factors need to be identified.

Dietary factors have been thought to account for about 30% of cancers in Western countries, making diet second only to tobacco consumption as a preventable cause of cancer (3). The role of dietary factors on the risk of bladder cancer is clearly plausible, as most substances or metabolites are excreted through the urinary tract and are consequently in direct contact with the mucosa of the bladder. Eggs are an important source of protein and fat and are widely consumed worldwide. A high intake of eggs has been associated with increased risk of several cancers (4–6), although other studies reported no association (7,8) or even decreased risk (9). Associations between bladder cancer incidence and intake of eggs have also been investigated, yielding inconclusive results.

The purpose of the present study was to investigate the association between bladder cancer and eggs intake. We conducted a meta-analysis of all published studies with greater statistical power to provide summary risk estimates for bladder cancer in relation to egg consumption.

## Materials and methods

### Database search

Eligible studies were identified by searching the PubMed database for relevant epidemiological studies of egg consumption in relation to the risk of bladder cancer dating from between January 1, 1980 and December 31, 2011. Additional publications identified by hand-searching of reference lists were also included. For computer searches, we used the following terms in any field: ‘eggs’ or ‘egg’ or ‘meat’ or ‘meats’ or ‘animal products’ combined with ‘bladder cancer’ or ‘urothelial cancer’. Studies were included in the meta-analyses if they presented risk estimates with corresponding 95% confidence intervals (CIs) from a cohort or case-control study in English language on the association between egg intake and incidence of bladder cancer. We also included articles evaluating the risk of urinary tract cancer with egg consumption, as the majority of cancers in the urinary tract are urothelial cancers, and bladder cancer comprises almost 90% cases of cancer in the lower urinary tract.

### Study analysis

The following pieces of information were extracted from the included studies: the name of the first author, the year of publication, the country in which the study was conducted, study design, year of follow-up (cohort studies) or data collection (case-control studies), sample size, anatomical site of the neoplasm, risk estimates with corresponding 95% CIs for highest vs. lowest level of egg consumption, exposure assessment and range of exposure, and adjusted covariates. We used risk estimates (REs) as the measure of the association in cohort and case-control studies. Crude (unadjusted) and adjusted REs were used for meta-analysis. Adjusted REs were extracted directly from the original reports. For the crude RE analysis, we extracted the number of cases and controls for the case-control studies and the number of cases and the person-years in the cohort studies, and calculated them from a two by two table.

### Statistical analysis

We estimated a pooled RE with 95% CI based on fixed- and random-effects models depending on the heterogeneity of the analysis. Statistical heterogeneity among studies included in the meta-analysis was assessed using the Q (10) and I^2^ statistics (11). Statistically significant heterogeneity was defined as p<0.05. Publication bias was assessed by the funnel plot and Egger’s test (12). Statistically significant publication bias was defined as p<0.05. Statistical analyses were performed using Stata 11.0 (Stata Corp, College Station, TX, USA).

## Results

### Study characteristics

**Figure 1:**
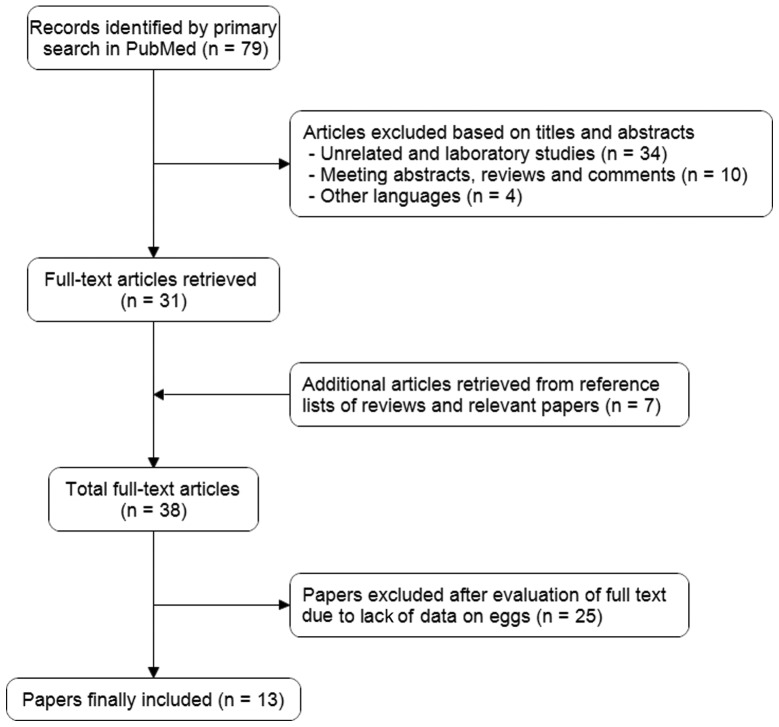
Process of study selection.

Fig. 1 shows the process of identifying and selecting studies. We finally included 13 articles that examined the risk of bladder cancer with egg intake published between January 1980 and December 2011, including four cohort, seven hospital-based and two population-based case-control studies (13–25). Six of these studies were conducted in Europe (13–15,22,23,25), one in the US (16), two in Uruguay (19,24), and four in Japan (17,18,20,21). Five studies included neoplasms of the urinary tract as cases (13,15,16,20,21). Table I presents the basic characteristics of each study included in our meta-analysis.

### Meta-analysis

**Figure 2:**
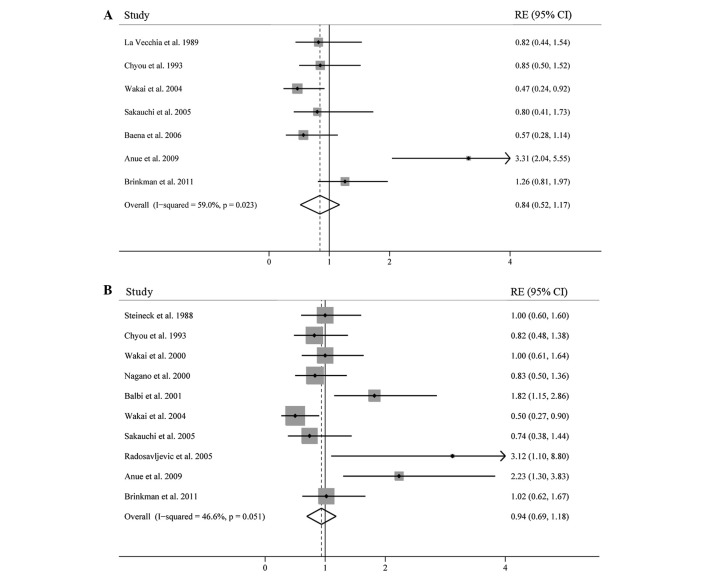
Forest plots showing risk estimates from case-control and cohort studies estimating the association between egg consumption and risk for bladder cancer using (A) crude data and (B) adjusted data.

We first calculated the summary RE for the highest vs. lowest category of egg consumption using the crude data; the pooled RE was 0.84 (95% CI, 0.52–1.12) in a random effects analysis (Fig. 2A). There was a statistically significant heterogeneity across studies (I^2^=66.6%; p=0.023). After excluding one study by Anue *et al*, which reported the highest point estimates, the p-value for heterogeneity among these studies was no longer statistically significant (I^2^=20.5%, p=0.279), and a significant inverse association was observed between egg consumption and bladder cancer risk (RE=0.73; 95% CI, 0.51–0.95).

**Figure 3:**
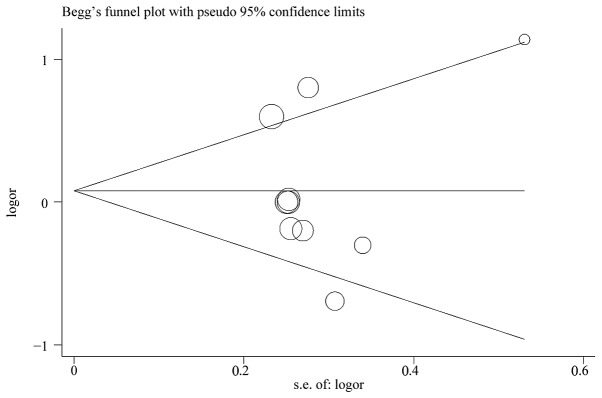
Funnel plot of egg consumption and bladder cancer risk.

However, when using the adjusted data, high egg consumption was not associated with a reduction in risk of bladder cancer (RE=0.94; 95% CI, 0.69–1.18) (Fig. 2B). There was a weak heterogeneity among all studies combined (I^2^=46.6%; p=0.051). To evaluate the stability of the results, we also performed a sensitivity analysis, which removed one study at a time. This analysis confirmed the stability of our results. No indication of publication bias was detected from either visualization of funnel plot (Fig. 3) or Egger’s test (p=0.883).

### Results of the subgroup analyses by study design, study population, egg cooking method and assessment are shown in Table II

In the subgroup analyses by study design, neither cohort studies (RE=0.85; 95% CI, 0.61–1.08) nor case-control studies (RE=1.17; 95% CI, 0.66–1.68) showed that egg intake was related to decreased bladder cancer risk. There was no evidence of heterogeneity among cohort studies, but some evidence among case-control studies. When subgroup analysis was conducted by study population, we found a statistically significant protective effect of egg consumption on bladder cancer for Japanese [including one study of Japanese in Hawaii (16); RR=0.72, 95% CI, 0.53–0.91], whereas a significantly increased risk was observed Uruguayans (RR=1.95, 95% CI, 1.24–2.66), and no association was found in Western countries (RR=1.19; 95% CI, 0.77–1.60). A subgroup analysis was also performed according to the assessment method of egg consumption. A statistically significant association was observed among studies using self-administered questionnaire techniques (RR=0.76; 95% CI, 0.55–0.97) but not among studies using interviews (RR=1.30; 95% CI, 0.77–1.84). In addition, it was noted that bladder cancer appeared to have a stronger positive association with fried egg intake (RR=2.00; 95% CI, 1.23–2.77), but not with boiled eggs.

## Discussion

Eggs are one of nature’s most nutritious foods, with low levels of saturated fat and high levels of protein. Although various health concerns are associated with egg consumption, it remains a popular ingredient in cooking worldwide. We systematically reviewed published epidemiological studies on the association between egg intake and the risk of bladder cancer. To our knowledge, this is the first meta-analysis to evaluate the relationship between them.

Egg consumption has not been studied as thoroughly as the consumption of meat and dairy products in relation to cancer risk. The most convincing evidence points to egg consumption as increasing risk for colorectal cancer (24,26,27). Several studies also found a positive association between egg intake and cancers of the oral cavity and pharynx (4,28,29). Recently, a large cohort study calculated that men who consumed 2.5 or more eggs per week had an 81% increased risk of lethal prostate cancer compared with men who consumed fewer than 0.5 eggs per week (30). The biological mechanism by which eggs have a detrimental effect on cancer risk possibly involves the high cholesterol content in egg yolk. Hu *et al* (31) found that high cholesterol intake is linked to increased risk of various cancers. Cholesterol is a precursor of steroid hormones, and accumulation of cholesterol in cells may affect prostate cancer risk through the formation of androgen. Alterations in cholesterol level could also contribute to cellular inflammation (32), which is a critical component of tumor progression.

In the present study, we first found that egg consumption was significantly associated with reduced risk of bladder cancer when using unadjusted estimates. However, this association became insignificant when models were adjusted for potential confounding variables. In subgroup analyses, the pooled analysis from the cohort studies, which have the advantage of being less vulnerable to selection and recall bias than case-control studies, also suggested no association. When subgroup analysis was conducted by egg cooking methods, we found a strong positive association between fried egg intake and bladder cancer risk, although the analysis was combined from only two studies. The elevated risk could be explained by formation of heterocyclic amines, which are known to be involved in bladder carcinogenesis by occupational exposure and smoking, during high-temperature cooking of eggs. We also observed a significantly negative association among studies using self-administered questionnaires but no association among studies using interviewing techniques. This contrast may be a consequence of response bias due to different assessment techniques or to chance alone. Notably, in subgroup analysis by study population, we noted a significant association of egg consumption with decreased risk of bladder cancer ws for Japanese, and this result was somewhat homogeneous (p=0.485; I^2^=0). However, increased risk was observed in Uruguayans. We are currently not able to identify a plausible explanation for the difference. A possible role of ethnic differences in genetic backgrounds might be taken into account.

Our study has several important limitations. First, the number of studies included was limited and we did not search for unpublished studies or original data, and therefore potential publication bias might influence the findings. However, no publication bias was indicated visually or in formal statistical testing. Second, residual confounders are always of concern in observational studies. Although the majority of included studies was adjusted for a wide range of potential confounders for bladder cancer, we were unable to exclude the possibility that other unmeasured or inadequately measured factors confounded the true association. Third, smoking is one of the major risk factors of bladder cancer, but we are unable to conduct stratified analyses adjusted by smoking, due to the lack of sufficient data from the included studies.

In summary, we did not find a significant association between overall egg intake and bladder cancer risk. However, this association varied significantly across different populations. Our findings have significant public health implications for high egg consumption worldwide. Given the small number of studies included in this meta-analysis, large prospective studies are required to confirm this association.

## Figures and Tables

**Table I:** Study characteristics of the published cohort and case-control studies on egg intake and bladder cancer.

Authors, year (ref.)	Study design	Country	Study period	Cases/subjects	Location of neoplasm	Egg consumption	RR (95% CI)	Variables of adjustment	Assessment
Steineck *et al*, 1988 (13)	Cohort	Sweden	1968–1982	80/16,477	Urothelial	Ever vs. never	1.0 (0.6–1.6)	Age, gender and smoking	Questionnaire
La Vecchia *et al*, 1989 (14)	HCC	Italy	1985–1987	163/344	Bladder	The highest vs. the first tertile	Unadjusted: 0.82 (0.44–1.54)	None	Interview
Steineck *et al*, 1990 (15)	PCC	Sweden	1985–1987	326/719	Urothelial	Weekly vs. less frequently	Boiled: 1.1 (0.7–1.8) Fried: 1.8 (1.0–3.1)	Age, gender and smoking	Questionnaire
Chyou *et al*, 1993 (16)	Cohort	USA	1965–1991	96/7,090	Lower urinary tract	5 times/week vs. ≤ once/week	Unadjusted: 0.85 (0.5–1.52) Males: 0.82 (0.48–1.38)	Age, smoking	Both methods
Nagano *et al*, 2000 (17)	Cohort	Japan	1979–1993	114/38,540	Bladder	≥ 5 times/week vs. ≤ once/week	0.83 (0.5–1.36)	Age, gender, radiation dose, smoking status, education level, body mass index and calendar time	Questionnaire
Wakai *et al*, 2000 (18)	HCC	Japan	1996–1999	297/592	Bladder	The highest vs. the first quartile	1.0 (0.61–1.64) Men: 0.89 (0.52–1.52)	Age, gender, smoking and occupational history as a cook	Interview
Balbi *et al*, 2001 (19)	HCC	Uruguay	1998–1999	144/720	Bladder	The highest vs. the first tertile	1.82 (1.15–2.86) Boiled:1.54 (0.96–2.46) Fried: 2.24 (1.37–3.66)	Age, gender, residence, urban/rural status, education, body mass index, tobacco smoking, *mate* drinking, and total calories	Interview
Wakai *et al*, 2004 (20)	HCC	Japan	1994–2000	124/744	Urothelial	≥ 5 times/week vs. ≤1–3 times/wk	Unadjusted: 0.47 (0.24–0.92) Adjusted: 0.5 (0.27–0.9)	Age, gender, year of first visit and cumulative consumption of cigarettes	Questionnaire
Sakauchi *et al*, 2005 (21)	Cohort	Japan	1988–1997	115/65,184	Urothelial	≥3–4 times/week vs. 1–2 times/month	Unadjusted: 0.8 (0.41–1.73) Adjusted: 0.74 (0.38–1.44)	Age, gender and smoking	Questionnaire
Radosavljević *et al*, 2005 (22)	HCC	Serbia	1997–1999	130/260	Bladder	The highest vs. the first tertile	Adjusted: 3.12 (1.1–8.8)	All variables that independently contributed to risk for bladder cancer	Interview
Baena *et al*, 2006 (23)	HCC	Spain	Not mentioned	74/163	Bladder	≥ 4 times/week vs. never	0.57 (0.28–1.14)	None	Interview
Anue *et al*, 2009 (24)	HCC	Uruguay	1996–2004	254/2,371	Bladder	≥ 4 times/week vs. never	2.23 (1.3–3.83)	Age, gender, residence, education, income, interviewer, smoking status, age at starting smoking, cigarettes per day, years since quitting smoking, duration of smoking, alcohol intake, intake of fruits and vegetables, grains, dairy foods, total meat, other fatty foods, *mate* drinking status, energy intake, BMI, men/women	Interview
Brinkman *et al*, 2011 (25)	PCC	Belgium	1999–2004	200/486	Bladder	The highest vs. the first tertile	1.02 (0.62–1.67)	Gender, age, smoking status, number of cigarettes smoked per day, number of years smoking, occupational exposure to PAHs or aromatic amines and energy intake	Questionnaire

HCC, hospital-based case-control studies; PCC, population-based case-control studies.

**Table II:** Summary of risk estimates of egg intake with bladder cancer by study design, exposure assessment, and geographical region.

Subgroup	Number of studies (reference)	Pooled RR (95% CI)	Q-test for heterogeneity P value (*I^2^* score)
Study design			
Cohort studies	4 (13,16,17,21)	0.85 (0.61, 1.08)	0.911 (0)
Case-control studies	9 (14,15,18–20,22–25)	1.17 (0.66, 1.68)	0.006 (69.4%)
Exposure assessment			
Interview	6 (13,15,17,20,21,25)	1.30 (0.77, 1.84)	0.076 (52.8%)
Mailed questionnaire	7 (14,16,18,19,22–24)	0.76 (0.55, 0.97)	0.517 (11.7%)
Geographical region			
Western studies	5 (13–15,22,23,25)	1.19 (0.77, 1.60)	0.267 (24.0%)
Japanese studies	5 (16–18,20,21)	0.72 (0.53, 0.91)	0.485 (0)
Uruguayan studies	2 (19,24)	1.95 (1.24, 2.66)	0.599 (0)
Cooking methods			
Fried eggs	2 (15,19)	2.00 (1.23, 2.77)	0.579 (0)
Boiled eggs	2 (15,19)	1.19 (0.69, 1.70)	0.24 (27.5%)

## References

[1] Genkinger JM, Hunter DJ, Spiegelman D (2006). A pooled analysis of 12 cohort studies of dietary fat, cholesterol and egg intake and ovarian cancer. Cancer Causes Control.

[2] Takeyama Y (2005). Dietary intake as a risk factor for pancreatic cancer in Japan: high cholesterol and low vitamin C diet. J Gastroenterol.

[3] Key TJ, Schatzkin A, Willett WC, Allen NE, Spencer EA, Travis RC (2004). Diet, nutrition and the prevention of cancer. Public Health Nutr.

[4] Franceschi S, Favero A, Conti E (1999). Food groups, oils and butter, and cancer of the oral cavity and pharynx. Br J Cancer.

[5] Nishimoto IN, Hamada GS, Kowalski LP (2002). Risk factors for stomach cancer in Brazil (I): a case-control study among non-Japanese Brazilians in Sao Paulo. Jpn J Clin Oncol.

[6] Marchand JL, Luce D, Goldberg P, Bugel I, Salomon C, Goldberg M (2002). Dietary factors and the risk of lung cancer in New Caledonia (South Pacific). Nutr Cancer.

[7] Ito LS, Inoue M, Tajima K (2003). Dietary factors and the risk of gastric cancer among Japanese women: a comparison between the differentiated and non-differentiated subtypes. Ann Epidemiol.

[8] Chow WH, Schuman LM, McLaughlin JK (1992). A cohort study of tobacco use, diet, occupation, and lung cancer mortality. Cancer Causes Control.

[9] De Stefani E, Boffetta P, Ronco AL (2005). Dietary patterns and risk of cancer of the oral cavity and pharynx in Uruguay. Nutr Cancer.

[10] DerSimonian R, Laird N (1986). Meta-analysis in clinical trials. Control Clin Trials.

[11] Higgins JP, Thompson SG, Deeks JJ, Altman DG (2003). Measuring inconsistency in meta-analyses. BMJ.

[12] Begg CB, Mazumdar M (1994). Operating characteristics of a rank correlation test for publication bias. Biometrics.

[13] Steineck G, Norell SE, Feychting M (1988). Diet, tobacco and urothelial cancer. A 14-year follow-up of 16,477 subjects. Acta Oncol.

[14] La Vecchia C, Negri E, Decarli A, D’Avanzo B, Liberati C, Franceschi S (1989). Dietary factors in the risk of bladder cancer. Nutr Cancer.

[15] Steineck G, Hagman U, Gerhardsson M, Norell SE (1990). Vitamin A supplements, fried foods, fat and urothelial cancer. A case-referent study in Stockholm in 1985–87. Int J Cancer.

[16] Chyou PH, Nomura AM, Stemmermann GN (1993). A prospective study of diet, smoking, and lower urinary tract cancer. Ann Epidemiol.

[17] Nagano J, Kono S, Preston DL (2000). Bladder-cancer incidence in relation to vegetable and fruit consumption: a prospective study of atomic-bomb survivors. Int J Cancer.

[18] Wakai K, Takashi M, Okamura K (2000). Foods and nutrients in relation to bladder cancer risk: a case-control study in Aichi Prefecture, Central Japan. Nutr Cancer.

[19] Balbi JC, Larrinaga MT, De Stefani E (2001). Foods and risk of bladder cancer: a case-control study in Uruguay. Eur J Cancer Prev.

[20] Wakai K, Hirose K, Takezaki T (2004). Foods and beverages in relation to urothelial cancer: case-control study in Japan. Int J Urol.

[21] Sakauchi F, Mori M, Washio M (2005). Dietary habits and risk of urothelial cancer incidence in the JACC Study. J Epidemiol.

[22] Radosavljevic V, Jankovic S, Marinkovic J, Dokic M (2005). Diet and bladder cancer: a case-control study. Int Urol Nephrol.

[23] Baena AV, Allam MF, Del Castillo AS (2006). Urinary bladder cancer risk factors in men: a Spanish case-control study. Eur J Cancer Prev.

[24] Aune D, De Stefani E, Ronco AL (2009). Egg consumption and the risk of cancer: a multisite case-control study in Uruguay. Asian Pac J Cancer Prev.

[25] Brinkman MT, Buntinx F, Kellen E (2011). Consumption of animal products, olive oil and dietary fat and results from the Belgian case-control study on bladder cancer risk. Eur J Cancer.

[26] Le Marchand L, Wilkens LR, Hankin JH, Kolonel LN, Lyu LC (1997). A case-control study of diet and colorectal cancer in a multiethnic population in Hawaii (United States): lipids and foods of animal origin. Cancer Causes Control.

[27] Steinmetz KA, Potter JD (1994). Egg consumption and cancer of the colon and rectum. Eur J Cancer Prev.

[28] Zheng W, Blot WJ, Shu XO (1992). Diet and other risk factors for laryngeal cancer in Shanghai, China. Am J Epidemiol.

[29] Levi F, Pasche C, La Vecchia C, Lucchini F, Franceschi S, Monnier P (1998). Food groups and risk of oral and pharyngeal cancer. Int J Cancer.

[30] Richman EL, Kenfield SA, Stampfer MJ, Giovannucci EL, Chan JM (2011). Egg, red meat, and poultry intake and risk of lethal prostate cancer in the prostate-specific antigen-era: incidence and survival. Cancer Prev Res (Phila).

[31] Hu J, La Vecchia C, de Groh M, Negri E, Morrison H, Mery L (2012). Dietary cholesterol intake and cancer. Ann Oncol.

[32] Ferretti G, Bacchetti T, Negre-Salvayre A, Salvayre R, Dousset N, Curatola G (2006). Structural modifications of HDL and functional consequences. Atherosclerosis.

